# Cyclin D1 mediated by the nuclear translocation of nuclear factor kappa B exerts an oncogenic role in lung cancer

**DOI:** 10.1080/21655979.2022.2043099

**Published:** 2022-03-04

**Authors:** Xin Wang, Xiaoping Liu, Yanxia Yang, Daowen Yang

**Affiliations:** a Department of Respiratory and Critical Care Medicine, Second People’s Hospital of Gansu Province & Northwest University for Nationality, Lanzhou, Gansu, China; b Department 1 of Lung Disease of TCM, China-Japan Friendship Hospital, Beijing, Chaoyang, China

**Keywords:** CCND1, NF-κB, the PI3K/AKT pathway, lung cancer, nuclear translocation

## Abstract

The relevance of cyclin D1 (CCND1) has been implicated in lung cancer progression. Nevertheless, the mechanism by which CCND1 supports lung cancer development is yet to be expounded. Here, we established that CCND1 is overexpressed in clinical lung cancer specimens and various lung cancer cells. Importantly, CCND1 overexpression enhanced lung cancer cell proliferation, invasion and migration, and arrested the cell cycle at the S phase. *In vivo*, overexpression of CCND1 promoted lung cancer growth and metastasis. The nuclear translocation of nuclear factor kappa B (NF-κB) promoted p65 protein expression and CCND1 transcription. Meanwhile, PI3K/AKT pathway activity was significantly reduced when NF-κB nuclear translocation was decreased. PI3K/AKT pathway activity was significantly elevated upon CCND1 overexpression. Inhibition of PI3K/AKT pathway activity or suppression of NF-κB translocation in cells with high CCND1 expression was found to significantly reduce the activity of lung cancer cells *in vitro* and *in vivo*. Our data revealed that NF-κB/CCND1/PI3K/AKT axis could act as a prospective diagnostic biomarker and a therapeutic option for lung cancer.

## GRAPHICAL ABSTRACT

**Figure:**
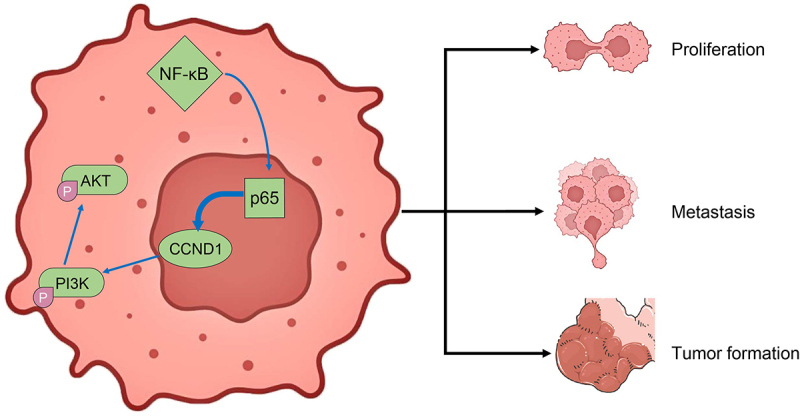

## Introduction

According to the latest global statistics in 2020, 2.2 million people and 1.8 million people were diagnosed as lung cancer and died from it, respectively [1]. As a consequence, lung cancer represents the most frequent contributor to cancer-associated deaths globally, with the 5-year survival rates varying from 4% to 17% depending on stage and regional disparities [2]. Rates continue to rise in low- and middle-income countries, and deaths associated with lung cancer can be averted through tobacco control measures [3]. Still, it is essential to clarify the mechanisms behind tumorigenesis and progression of lung cancer, and to identify prospective targets for lung cancer control.

Cyclins coordinate with their cyclin-dependent kinases to drive cells from one phase of the cell cycle to another [4]. Among them, cyclin D1 (CCND1) is a main regulating factor in cell cycle (G1 phase) encoded by chromosome 11q13 CCND1 gene, firstly discovered in 1991 [5]. The oncogenic roles of the CCND1 have been displayed in many human cancers, including thyroid, hepatocellular, colon, and prostate cancers [^6–9^]. Transcription factor activity is changed in a large repertoire of cancers via multiple direct mechanisms, involving chromosomal translocations, gene amplification or deletion, and alteration of expression, along with indirect mechanism through non-coding DNA mutations [10]. For instance, the transcription of CCND1 has been described to be regulated by HMG box protein 1 in cancers [11]. In the present study, we set to dissect the possible upstream transcription factor of CCND1 in lung cancer. The nuclear factor kappa B (NF-κB) family is characterized by their distinctive structure, an N-terminal Rel homology domain, and the p65 (RelA), RelB and c-Rel proteins boast a C-terminal transactivation domain which interacts with the transcription machinery that enhances gene transcription [12]. NF-κB is a transcription factor that promotes expression of more than 200 genes involved in varied processes, such as cell survival, adhesion, inflammatory response, differentiation and growth, and induction of NF-κB upregulated the expression patterns of its responsive genes in cancer cells, including lung cancer cells [13]. However, whether there is a direct binding relationship between NF-κB and CCND1 in lung cancer has not been reported. CCND1 has been reported to play key roles in cell biology, including cell proliferation and growth regulation, mitochondrial activity modulation, and cell migration control in oral carcinogenesis [14]. Thus, we hypothesize that the function of CCND1 in lung cancer cell proliferation and migration was associated with its transcription regulated by NF-κB. In the present study, we aimed to evaluate the overexpression of CCND1 in lung cancer cell growth *in vitro* and *in vivo* and explore the molecular mechanisms associated with NF-κB.

## Materials and methods

### Database analysis

Lung cancer datasets GSE32863 and GSE108055 were downloaded from the GEO database (https://www.ncbi.nlm.nih.gov/geo/). The R script (version 4.0.1, http://cran.r-project.org/) was used for bioinformatics analysis. The LIMMA package (Bioconductor, Seattle, WA, USA) was used to identify significant changes in gene expression between cancer and normal samples and to evaluate differentially expressed genes using Bayesian analysis. p values <0.05 and |Foldchange| >2 were considered as significance thresholds. The ClusterProfiler package (Bioconductor) was used to perform the gene ontology (GO) enrichment analysis, and the annotation information was downloaded from the GO database (http://www.bioconductor.org/packages/release/data/annotation/) via the GOplot package (Bioconductor) for visualization of pathways. The promoter sequence of CCND1 was obtained by UCSC (https://genome.ucsc.edu/index.html), and the binding relationship between p65 and the CCND1 promoter sequence was tested.

### Tissue samples

All participators were informed about the study procedure and have provided consent to participate. The study protocol was permitted by the Ethics Committee of Second People’s Hospital of Gansu Province (approval number: 201316). The lung cancer and paired adjacent tissues (more than 5 cm from the tumor tissue) were clinically collected from 68 patients with lung cancer who were admitted to Second People’s Hospital of Gansu Province between January 2014 and February 2016. The tissues were snap-frozen in liquid nitrogen instantly after surgical resection and stored at −80°C for further RNA isolation.

### RT-qPCR assays

Total RNA from cells and tissues was extracted using RNAiso Plus Trizol Reagent (Takara Biotechnology Ltd., Dalian, Liaoning, China). Total RNA (1 μg) was applied for cDNA synthesis, and reverse transcription was implemented using the PrimeScript RT kit (TaKaRa) at 37°C for 15 min and 85°C for 5 s. Changes in gene expression were detected using the SYBR PCR Master Mix kit (TaKaRa). Data were calculated using the comparative threshold cycle (Ct) method [15], and the endogenous control for mRNA was glyceraldehyde 3-phosphate dehydrogenase (GAPDH) [16]. All primary sequences were synthesized by RiboBio (Guangzhou, Guangdong, China). The primer sequences were as follows: CCND1 (forward 5’-CTGATTGGACAGGCATGGGT-3’ and reverse 5’-GTGCCTGGAAGTCAACGGTA-3’); GAPDH (forward 5’-AGAAGGCTGGGGCTCATTTG-3’ and reverse 5’-AGGGGCCATCCACAGTCTTC −3’).

### Western blot

Western blot analysis was performed as described [17]. The cells were lysed with radio immunoprecipitation assay buffer (Thermo Fisher scientific) containing proteinase inhibitor, and equal amounts of protein were isolated by SDS-PAGE and transferred to nitrocellulose membranes (Millipore Corp, Billerica, MA, USA). The membranes were blocked in Tris-buffered saline with Tween containing 5% skimmed milk for 1 h at ambient temperature and incubated with the primary antibody at 4°C overnight, followed by incubation with the secondary antibody for 1 h at ambient temperature. Protein expression was detected based on ECL kits (Amersham Pharmacia, Piscataway, NJ, USA). The bands were exposed and observed using the Bio-Rad ChemiDoc XRS^+^ imaging system (Bio-Rad Laboratories, Hercules, CA, USA). The primary antibodies included rabbit antibodies against CCND1 (1:1000, ab16663, Abcam), p-p65 (1:800, ab76302, Abcam), PI3K (1:1000, ab32089, Abcam), p-PI3K (1:1,200, ab182651, Abcam) and mouse antibodies to AKT (1:700, 10176-2-AP, Proteintech Group), p-AKT (1:500, 66444-1-Ig, Proteintech Group) and GAPDH (1:1,000, ab8245, Abcam). The secondary antibodies included goat anti-rabbit IgG (1:5,000, ab205718, Abcam) and goat anti-mouse IgG (1:5,000, ab205719, Abcam).

### Cell culture and treatments

The human lung cancer cells A549, H358 and the human bronchial epithelial (BEAS-2B) cells were from the American Type Culture Collection (Manassas, VA, USA). PC9 was purchased Procell (Wuhan, Hubei, China), and PC13 from Immuno-Biological Laboratories (Tokyo, Japan). A549 cells were cultivated in DMEM (Thermo Fisher Scientific Inc., Waltham, MA, USA), and H358, PC9 and PC13 cells were incubated in Roswell Park Memorial Institute (RPMI)-1640 medium (Thermo Fisher scientific). About 100 U/mL penicillin, 100 U/mL streptomycin and 10% fetal bovine serum (FBS) were supplemented to both media. All the cells were maintained with 5% CO_2_ at 37°C.

CCND1 overexpression (CCND1-OE) provided by GenePharma (Shanghai, China) was inserted into the expression vector GV248 (Shanghai Genechem Co., Ltd., Shanghai, China). The cells were transfected with Lipofectamine 2000 (Thermo fisher scientific). Stably transfected cells were screened under puromycin (Sigma-Aldrich Chemical Company, St Louis, MO, USA). Cell colonies were obtained by limited dilution method.

NF-κB nuclear translocation was inhibited using the NF-κB inhibitor SN50 (213546–53-3, MedChemExpress, Monmouth Junction, NJ, USA) [18]. The cells were seeded into 96-well plates (7,000 cells/well) and incubated for 24 h, and 18 µM SN50 was added to the culture medium. The cells were grown in a humidified incubator at 37°C with 5% CO_2_ for another 24 h before subsequent experiments. The PI3K/AKT pathway activity was inhibited by the inhibitor PI3K-IN-1 (HY-12068, MedChemExpress), and the cells were treated for 24 h and with 25 μM PI3K-IN-1 in culture medium at 37°C and 5% CO_2_ for 24 h for following experiments.

### Ethynyl deoxyuridine (EdU) incorporation assays

Cell proliferation experiments were performed using the CellLight EdU Apollo 567 *in vitro* imaging kit (RiboBio). The cells were plated into 96-well plates at 8 × 10^3^ cells at 48 h post-transfection, incubated in 50 mol/L EdU solution at 37°C for 2 h, fixed with 4% paraformaldehyde for 30 min, and then treated with 0.5% Triton X-100 for 10 min. The cells were then incubated with 100 mL Apollo reaction cocktail for 30 min, followed by incubation with 5 mL Hoechst 33342 solution for 30 min to stain nuclei. The cells were photographed using a fluorescence microscope (Olympus Optical Co., Ltd., Tokyo, Japan), and the cell proliferation rate was assessed by calculating the percentage of EdU-positive cells.

### Transwell assays

In the migration experiment [19], 5 × 10^4^ cells were resuspended in serum-free medium (Thermo Fisher scientific) and placed in the apical chamber (24-well insert, 8 μm, Corning Glass Works, Corning, N.Y., USA). Growth medium plus 10% FBS was used as chemoattractant in the basolateral chamber. After 24 h of incubation, the plates were fixed with 4% paraformaldehyde (Santa Cruz Biotechnology Inc., Santa Cruz, CA, USA) and stained with 0.5% crystal violet (Sangon, Shanghai, China). The images of stained cells were collected under a microscope (Olympus), and five random fields were selected for counting. For the invasion assay, the procedure was similar to the migration assay, except that the Transwell membranes were additionally precoated with Matrigel (BD Biosciences, San Jose, CA, USA).

### Flow cytometry

The cells were seeded in 6-well plates (1 × 10^6^ cells/well) and permeabilized with 75% ethanol overnight at 4°C. The cells were centrifuged at 1,000 g for 5 min, and incubated with 10 μL propidium iodide (PI, Sigma Aldrich) for 0.5 h at 37°C in darkness. Cell cycle changes were assessed by a flow cytometer (FACScan; BD Biosciences) [20].

### Immunofluorescence staining

The cells were seeded into 6-well plates and incubated for 24 h. After being fixed with 4% formaldehyde for 10 min, the cells were incubated with 1% BSA in PBS-tween for 1 h to block nonspecific protein-protein relationships. The cells were incubated with antibodies against p65 (1:100, ab32536, Abcam) at 4°C overnight, and then with green fluorescent-labeled secondary antibody (1:50, ab150077, Abcam) for 60 min at ambient temperature. 4’,6-diamidino-2-phenylindole (Solarbio, Beijing, China) was supplemented for a 15-min incubation at room temperature. Finally, an anti-fluorescence sealing solution (Solarbio) was added to prevent fluorescence decay, and the fluorescence of the cells was observed using a fluorescence confocal microscope (FV10i, Olympus).

### Chromatin immunoprecipitation (ChIP) assay

The stably growing cells were fixed with 4% paraformaldehyde for 10 min, ultrasonically treated for 2 h, and centrifuged at 13,000 rpm at 4°C for 5 min. The supernatants were incubated overnight at 4°C with a negative control antibody rabbit anti-IgG (ab109489, 1:100, Abcam) and a specific rabbit antibody to p65 (1:100, ab32536, Abcam). Afterward, Protein Agarose/Sepharose (Solarbio) was added to precipitate for 30 min, and then the supernatant was aspirated after centrifugation. The nonspecific complexes were washed with PBS, and the DNA was de-crosslinked overnight at 65°C, recovered by phenol/chloroform extraction and purification. RT-qPCR was performed to assess the enrichment of p65 [21].

### Enzyme linked immunosorbent assay (ELISA)

The transfected cells were lysed with RIPA buffer (Thermo Fisher scientific) with a proteinase inhibitor. The p65 level was measured using the NF-κB p65 Human ELISA Kit (KHO0371, Thermo Fisher scientific). The optical density (OD) value at 450 nm was measured using a BioTek optical plate reader (BioTek Instruments, Winooski, VT, USA) and converted to concentration (pg/mL) using a standard calibration curve.

### In vivo tumorigenesis and metastasis assays in nude mice

The Research Ethics Committee of Second People’s Hospital of Gansu Province approved the animal experiments (approval number: 201908). Thirty BALB/c nude mice (4 weeks old, 20 ± 2 g) were from Vital River (Beijing, China). A549 and PC13 cells (1 × 10^6^) were diluted in 200 μL PBS and 200 μL Matrigel (BD Biosciences) under sterile conditions and injected subcutaneously into anesthetized nude mice to establish a lung cancer xenograft model. The tumor volume of mice was measured at an interval of 4 days after injection of lung cancer cells, and the mice were euthanized 16 days later by an overdose of pentobarbital sodium (120 mg/kg). The tumor volume was calculated as 1/2 × the long axes × the short axes^2^ [22].

Thirty BALB/c nude mice were euthanized by an overdose of pentobarbital sodium at 120 mg/kg 45 days after tail vein injection of 1 × 10^6^ A549 and PC13 cells. Liver tissues were extracted for hematoxylin-eosin (HE) staining to observe the formation of metastases. HE staining kits were from Beyotime (Shanghai, China). The liver tissues were preserved after conventional paraffin embedding. The liver sections were dewaxed, stained with hematoxylin for 5 min, differentiated with 1% ethanol hydrochloride for 3 s, and stained with 5% eosin for 3 min. Observation of tissue sections was conducted under a fluorescence microscope (Olympus).

### Immunohistochemistry

Tumor tissues from mice were embedded in paraffin, dewaxed and rehydrated. Antigen retrieval was performed by incubating the slides in 10 mmol/L sodium citrate buffer and microwaving the samples for 20 min. After sealed with 3% H_2_O_2_ and 10% normal goat serum, the slides were incubated with a mouse monoclonal antibody against CCND1 (1:500, 60186-1-Ig, ProteinTech Group, Chicago, IL, USA) overnight at 4°C. The slides were incubated with biotin-coupled anti-mouse secondary antibody (1:1,000, ab205719, Abcam, Cambridge, UK) for 2 h at 37°C using an ABC kit (Vector Laboratories, Burlingame, CA, USA), followed by incubation with polymeric horseradish peroxidase reagents. The peroxidase activity was observed in diaminobenzidine (Vector Laboratories), and the sections were counter-stained with hematoxylin.

### Statistical analysis

All quantified data represented an average of at least triplicate samples. Data are presented as mean ± SD. Results were statistically analyzed using IBM SPSS v20.0 (IBM Corporation, Armonk, NY, USA) using unpaired *t*-test, one-way or two-way ANOVA, followed by Tukey’s multiple comparison tests. Gene enrichment analysis was carried out using Fisher’s exact test. Survival analysis was implemented using Kaplan-Meier method. Differences were considered statistically significant when *p* < 0.05.

## Results

In this study, we studied the biological role and the molecular mechanisms of CCND1 in lung cancer. CCND1 overexpression was found in lung cancer cells and tissues, and enhanced the cell proliferation, migration, invasion and cell cycle arrest of lung cancer cells. Additionally, the nuclear translocation of NF-κB enhanced the transcription of CCND1 to induce the activation of the PI3K/AKT signaling pathway. Thus, we revealed that CCND1, mediated by the nuclear translocation of NF-κB, promoted the progression of lung cancer through triggering PI3K/AKT signaling pathway.

### CCND1 is overexpressed in lung cancer tissues

**Figure 1.**
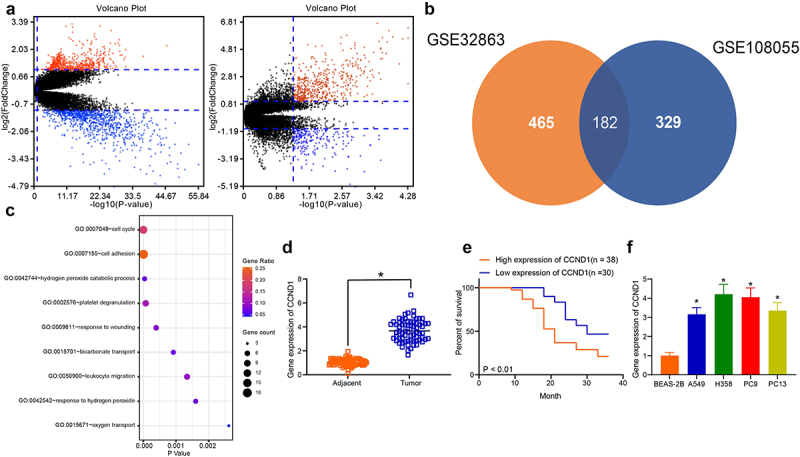
CCND1 expresses highly in lung cancer tissues and cells. (a) differentially expressed genes derived from bioinformatics analysis and volcano mapping; (b) the Venn map screening for differentially expressed genes in intersection; (c) GO enrichment analysis prediction for BP pathway analyzed by Fisher’s exact test; (d) RT-qPCR for CCND1 expression in lung cancer and adjacent tissues; (e) Kaplan-Meier analysis of patients differentially expressing CCND1 (differentiated by the median value); (f) RT-qPCR detection of CCND1 expression patterns in lung cancer cells. Error bars represent standard deviations of the means of three biological replicates. Values represent means ± SD. *p < 0.05. Results were analyzed by one-way ANOVA, followed by Tukey’s post hoc tests (panel F) or paired *t* test (panel D).

The GSE32863 dataset (n = 58) and the GSE108055 dataset (n = 64) were analyzed, and the volcanoes for differentially expressed genes were plotted (Figure 1a). The screened genes were overlaid by a Venn plot, and 182 genes were found to be differentially expressed in both datasets (Figure 1b). GO enrichment analysis was performed on the differentially expressed genes, and the enrichment term was biology process (BP). It was found that the differentially expressed genes were mainly enriched in cell cycle regulation (Figure 1c). For GO functional annotation of the genes enriched in this pathway, we noticed that CCND1, which is known to be one of the key proteins in cell cycle regulation, was differentially expressed in lung cancer. We speculated that the differential expression of CCND1 may be the reason why the BP in lung cancer is mainly regulated by the cell cycle. The gene expression of CCND1 between lung cancer and adjacent tissues examined by RT-qPCR revealed a 3-fold difference in mRNA expression (Figure 1d). The correlation between CCND1 expression patterns and survival of lung cancer patients was analyzed using Cox multiple regression, and the results showed that high CCND1 expression was related to dismal prognosis of patients (Figure 1e). Detection of CCND1 expression in lung cancer cells revealed that CCND1 was generally elevated in lung cancer cells (figure 1f). Through a series of experiments and analysis, we believe that CCND1 may be a key gene in the regulation of lung cancer, and has certain prognostic significance.

### CCND1 promotes lung cancer cell activity

**Figure 2.**
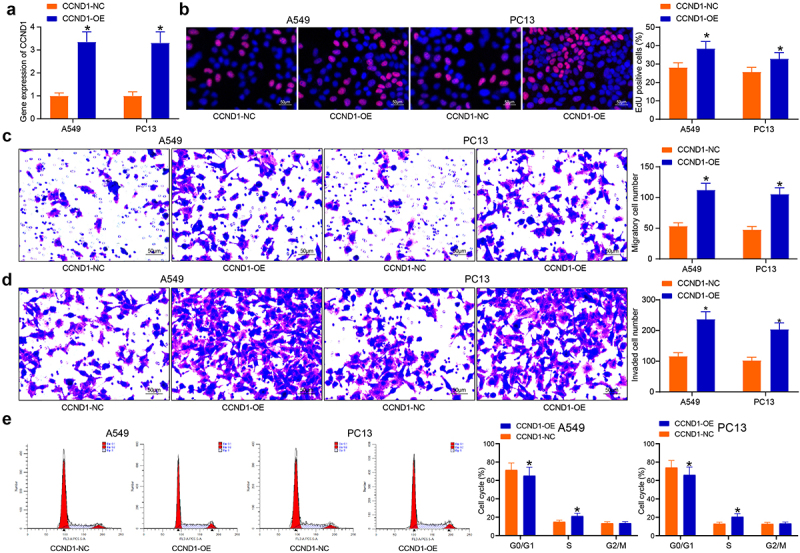
CCND1-overexpressing lung cancer cells show enhanced proliferation and S phase arrest. (a) validation of CCND1-OE transfection efficacy by RT-qPCR; (b) the proliferative activity of lung cancer cells examined by EdU; (c) cell migration activity examined by Transwell assay; (d) cell invasion activity examined by Transwell assay; (e) the cell cycle examined by flow cytometry. Error bars represent standard deviations of the means of three biological replicates. Values represent means ± SD. *p < 0.05. Results were analyzed by two-way ANOVA, followed by Tukey’s post hoc tests.

To explore the molecular function and mechanism of CCND1, A549 and PC13 cells stably overexpressing CCND1 were established (Figure 2a). The effect of CCND1 expression on the proliferation of lung cancer cells was firstly analyzed, and EdU staining of proliferating cells was followed by observation of the proportion of positive cells under fluorescence microscope to assess the cellular DNA replication. The promotion of CCND1 expression led to an increase in EdU-positive cells in A549 and PC13 cells and a significant increase in cell proliferation (Figure 2b). Analysis of the effects of CCND1 expression on lung cancer cell migration and invasion showed that CCND1-OE enhanced cell migration and invasion activities (Figure 2c,d). Because CCND1 is a key gene in the regulation of cell cycle, we examined the cycle changes of cells with high expression of CCND1 by flow cytometry. Upregulation of CCND1 contributed to a significant decrease in the G0/G1-phase, a significant augment in the proportion of S-phase cells, and no significant change in the proportion of G2/M-phase cells (Figure 2e).

### CCND1 promotes lung cancer development in vivo

**Figure 3.**
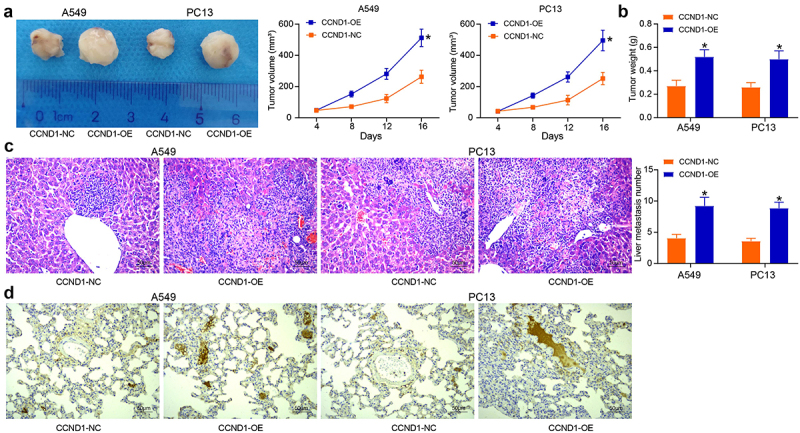
Overexpression of CCND1 promotes lung cancer growth *in vivo*. (a) tumor volume changes in mice with high expression of CCND1; (b) assessment of tumorigenic capacity by tumor weight; (c) HE staining for metastatic nodule formation in mouse liver tissues; (d) immunohistochemical detection of CCND1 protein in lung tissues. Error bars represent standard deviations of the means of three biological replicates. Values represent means ± SD. *p < 0.05. Results were analyzed by two-way ANOVA, followed by Tukey’s post hoc tests.

The cells overexpressing CCND1 were injected subcutaneously into mice. The changes in tumor volume in mice within 16 days were detected, and the growth curves were plotted. Subcutaneous xenografts were similar to a spherical shape, forming an intact pseudocapsule, and central tissue congestion and necrosis were found. Upregulation of CCND1 accelerated the growth rate and increased the growth volume of mouse xenograft tumors (Figure 3a). In addition, CCND1-OE elevated tumor weight in mice and supported the tumorigenicity of lung cancer cells *in vivo* after 16 days (Figure 3b). The effect of CCND1 on *in vivo* metastatic activity was also detected, and HE staining of liver tissues of mice showed that CCND1 upregulation significantly increased the number of liver metastatic nodules (Figure 3c). The protein expression of CCND1 in cancer tissues was assessed by immunohistochemistry, and the protein expression of CCND1 was found to be higher in cancer tissues with CCND1 overexpression (Figure 3d). Therefore, CCND1 promotes tumorigenicity and metastasis of lung cancer cells.

### CCND1 activity is mediated by the nuclear translocation of NF-κB

**Figure 4.**
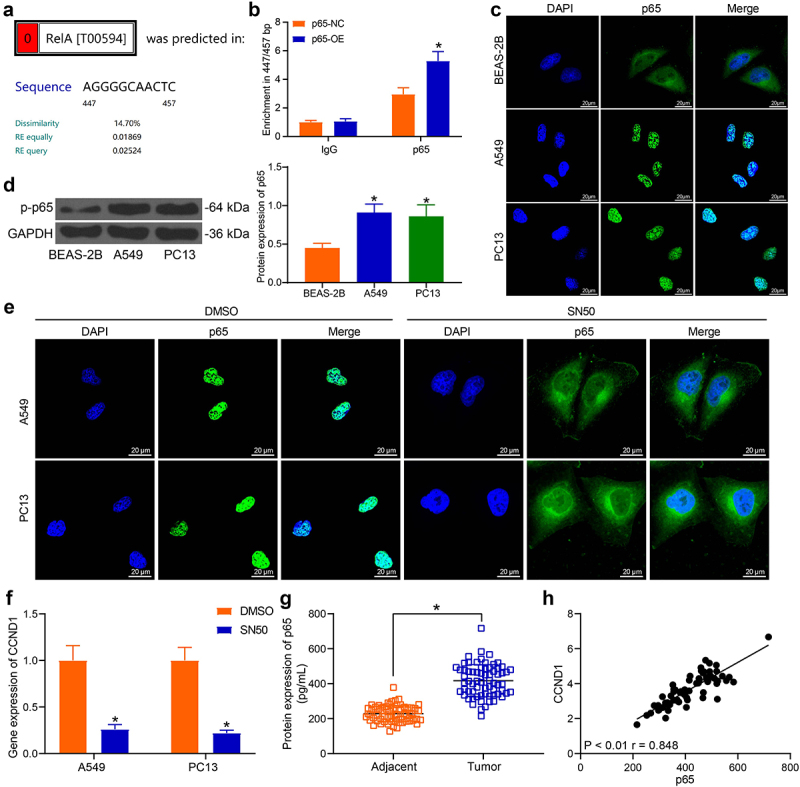
NF-κB upregulates CCND1 expression in lung cancer cells. (a) the binding sites of p65 to CCND1 was obtained from the bioinformatics analysis; (b) the binding of NF-κB to CCND1 validated using ChIP assay; (c) NF-κB nuclear translocation between cancer cells and normal cells detected by immunofluorescence; (d) Western blot detection of p-p65 protein levels in cells; (e) immunofluorescence detection of p65 protein after SN50 treatment; (f) CCND1 expression after SN50 treatment detected by RT-qPCR; (g) detection of p65 protein in cancer tissues and adjacent tissues from lung cancer patients by ELISA; H, Person’s correlation analysis of NF-κB and CCND1 expression in cancer tissues. Error bars represent standard deviations of the means of three biological replicates. Values represent means ± SD. *p < 0.05. Results were analyzed by paired *t* test (panel G), one-way (panel D) or two-way (panel B and F) ANOVA, followed by Tukey’s post hoc tests.

To explore the upstream gene of CCND1, we postulated that a transcription factor may mediate the transcription of CCND1. By analyzing genes in the cell cycle regulation as described above, we found that p65 acts as a transcription factor in the cells. The promoter sequence of CCND1 was obtained by UCSC, and the binding relationship between the promoter sequence of CCND1 and p65 was detected by ALEGN. The 447/457 bp region upstream of the CCND1 promoter contains a binding site for p65 (Figure 4a). The enrichment of p65 protein was significantly increased after overexpression of p65 in the 447/457 bp region upstream of the CCND1 promoter, indicating an immunoprecipitation relationship between p65 and the CCND1 promoter (Figure 4b). It has been reported that the nuclear translocation of p65, the subunit of NF-κB, activated NF-κB to promote CCND1 transcription [23]. We then speculated whether the transcription of CCND1 was mediated by the nuclear translocation of NF-κB. Therefore, fluorescence-labeled NF-κB in lung cancer cells and human bronchial epithelial (BEAS-2B) cells were observed under fluorescence microscopy. Compared with BEAS-2B cells where NF-κB protein was basically present in the cytoplasm, lung cancer cells A549 and PC13 showed a tendency of NF-κB nuclear localization (Figure 4c). The nuclear translocation of p65 between cancer cells and normal cells also contributed to the increase of p-p65 protein level by Western blot (Figure 4d).

In order to examine whether the nuclear translocation of NF-κB has an effect on CCND1 expression, we used the NF-κB inhibitor SN50, which can effectively inhibit the nuclear translocation of NF-κB, to treat lung cancer cells. The nuclear translocation of NF-κB was significantly repressed, and the protein in the nucleus was reduced by fluorescence analysis after SN50 treatment (Figure 4e). We found that the expression of CCND1 was also suppressed after the inhibition of NF-κB nuclear translocation (figure 4f). ELISA quantification in clinically collected lung cancer tissues validated that the expression of p65 was significantly boosted in lung cancer tissues (Figure 4g). Correlation analysis revealed a high degree of correlation between CCND1 and p65 expression in cancer tissues (Figure 4h).

### CCND1 mediates the PI3K/AKT pathway in lung cancer cells

**Figure 5.**
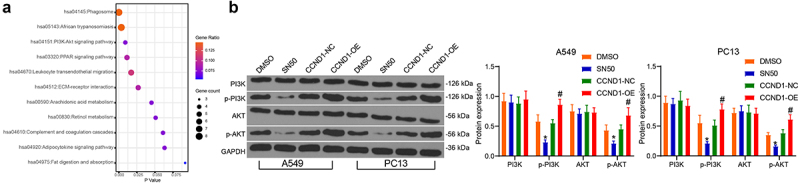
CCND1 mediates the PI3K/AKT pathway in lung cancer cells. (a) Fisher’s exact test of gene enrichment pathways by KEGG pathway analysis; (b) Western blot detection of changes in PI3K/AKT pathway activity in SN50 and CCND1-OE-treated cells. Error bars represent standard deviations of the means of three biological replicates. Values represent means ± SD. *p < 0.05, #*p* < 0.05. Results were analyzed by two-way ANOVA, followed by Tukey’s post hoc tests.

To probe the pathway activated by CCND1 in cell cycle regulation, we performed KEGG enrichment analysis of genes enriched in the cell cycle regulation. Most of the genes were found to be enriched in the PI3K/AKT pathway, in which NF-κB and CCND1 were also enriched (Figure 5a). The PI3K/AKT pathway was significantly blocked when NF-κB nuclear translocation was inhibited, and increased when CCND1 was highly expressed (Figure 5b). It was suggested that CCND1 may control the activity of lung cancer cells through mediating the PI3K/AKT pathway, but further validation was needed.

### Validation of the NF-κB/CCND1/PI3K/AKT axis in vitro

**Figure 6.**
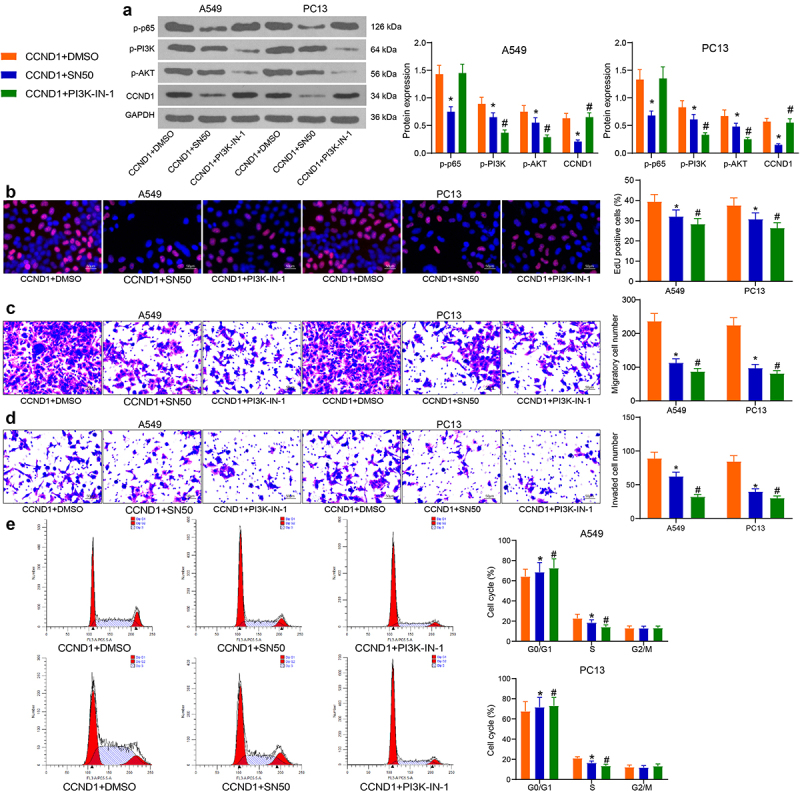
Depletion of NF-κB or the PI3K/AKT pathway abrogates the oncogenic roles of CCND1 on lung cancer cell proliferation and cell cycle progression. (a) the protein expression of CCND1 and the extent of p65, PI3K and AKT phosphorylation by Western blot; (b) the proliferative activity of lung cancer cells examined by EdU; (c) cell migration activity examined by Transwell assay; (d) cell invasion activity examined by Transwell assay; (e) the cell cycle examined by flow cytometry. Error bars represent standard deviations of the means of three biological replicates. Values represent means ± SD. *p < 0.05, #*p* < 0.05. Results were analyzed by two-way ANOVA, followed by Tukey’s post hoc tests.

The cells overexpressing CCND1 were further treated with SN50 or PI3K-IN-1, an inhibitor of PI3K/AKT pathway. The effect of the inhibitors was verified by Western blot detection of the CCND1 protein expression and the extents of p65, PI3K, and AKT phosphorylation in cells. The addition of SN50 in cells was found to decrease the extent of p65 phosphorylation in cells, with a consequent decrease in the protein content of CCND1, while PI3K-IN-1 decreased the extent of PI3K and AKT phosphorylation in cells (Figure 6a). SN50 and PI3K-IN-1 decreased the proliferative activity of the cells overexpressing CCND1 in EdU assay (Figure 6b). Detection of cell migration and invasion capacities revealed that inhibition of NF-κB nuclear translocation and PI3K/AKT pathway activity attenuated the migration activity of cells with high expression of CCND1 (Figure 6c) and reduced CCND1-OE-induced cell invasion capacity (Figure 6d). Finally, cell cycle changes were detected by flow cytometry, and inhibition of NF-κB nuclear translocation and PI3K/AKT pathway activity led to a significant decrease in the proportion of S-phase cells (Figure 6e).

### Validation of the NF-κB/CCND1/PI3K/AKT axis in vivo

**Figure 7.**
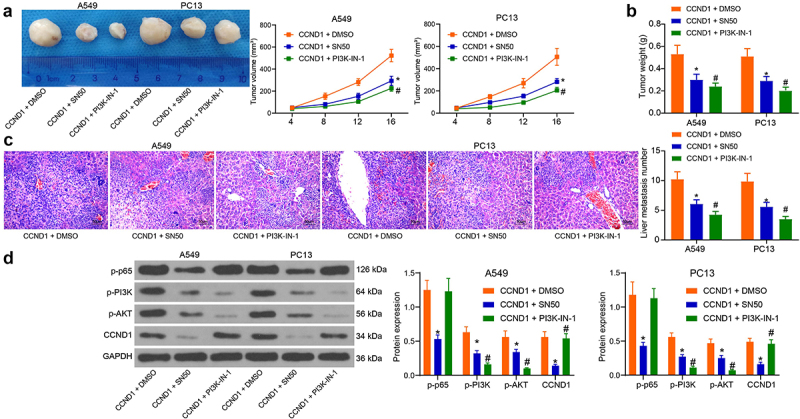
Depletion of NF-κB or the PI3K/AKT pathway abrogates the oncogenic roles of CCND1 on lung cancer cell progression and liver metastases. (a) tumor volume changes in mice; (b) assessment of tumorigenic capacity by tumor weight; (c) HE staining for metastatic nodule formation in mouse liver tissues; (d) protein expression changes in mouse xenograft tumor tissues examined using Western blot. Error bars represent standard deviations of the means of three biological replicates. Values represent means ± SD. *p < 0.05, #*p* < 0.05. Results were analyzed by two-way ANOVA, followed by Tukey’s post hoc tests.

The interrelationship between NF-κB/CCND1/PI3K/AKT was verified *in vivo*. When lung cancer cells were injected into mice, the declines in NF-κB activity and PI3K/AKT activity diminished the tumor volume and growth rate of mice (Figure 7a). Also, tumor weight was reduced by SN50 and PI3K-IN-1 (Figure 7b). After HE staining, the formation of liver metastatic nodules in mice was observed under the microscope, and the decrease of NF-κB and PI3K/AKT activities significantly inhibited the action of CCND1 to delay liver metastasis in mice (Figure 7c). Lastly, protein expression in tumor tissues was examined using Western blot. We verified the effect of CCND1 inhibition by NF-κB inhibitor SN50 in tumor tissues with changes in the pathway activity of PI3K/AKT (Figure 7d). We thus demonstrated the interrelationship between NF-κB, CCND1 and the PI3K/AKT pathway through a series of *in vitro* and *in vivo* experiments.

## Discussion

Through mining the two online mRNA datasets and GO analysis, we discovered that CCND1 is one of the overlaps, implying it as a possible target in lung cancer. Direct binding relationship between CCND1 and NF-κB in lung cancer was validated by immunofluorescence and ChIP assays in the current investigation. By manipulating CCND1 or/and NF-κB levels in lung cancer cells, we revealed that (1) overexpression of CCND1 resulted in cell proliferation and induced cell cycle arrest at the S phase; (2) overexpression of CCND1 promoted lung cancer progression and liver metastases; (3) Depletion of NF-κB and the following PI3K/AKT pathway in conjunction with CCND1 upregulation sufficed to reverse the oncogenic roles of CCND1. These findings corroborated that CCND1 mediated by NF-κB nuclear translocation serves as an oncogene in lung cancer cells via the PI3K/AKT pathway.

Previously, meta-analysis conducted by Binabaj *et al*. illustrated that the upregulation of CCND1 was tightly linked to poor prognosis in patients with head and neck cancer and could be a potential prognostic marker for this cancer type [24]. Moreover, CCND1 amplification in primary tumors and metastases predicted cancer-related death independently in bladder cancer [25]. Consistently, we observed that CCND1 was drastically elevated in lung cancer tissues relative to adjacent tissues (by 3-fold), which was linked to the poor survival of patients with lung cancer. As we mentioned above, CCND1 upregulation is responsible for the enhanced lung cancer progression and liver metastases *in vivo*. Overexpression of CCND1 contributed to rapid cell growth under conditions of impaired mitogenic signaling, bypass of major cellular checkpoints, and finally, neoplastic growth [26]. The tumor supporting role of CCND1 has been substantiated in lung cancer via a binding relation with microRNAs or long noncoding RNAs [^27–31^], indicating that the expression of CCND1 might be regulated by a myriad of mechanisms. Moreover, FXR, a transcription factor, has been suggested to be recruited to the CCND1 promoter and elevated its transcription in lung cancer [32]. Under the context of clear cell renal cell carcinoma, nuclear transcription factor Y binds directly to the promoter region of CCND1, thus transactivating its expression [33]. As a result, we postulated that another transcription factor exists to regulate the CCND1 transcription in lung cancer.

Tight association of NF-κB with tumorigenesis makes it a promising target for basic research and pharmaceutical industries, and many animal and cell models have revealed the significance of NF-κB in pathobiology of lung diseases [34]. NF-κB is participated in most if not all cellular events in tumor progression, including inflammation, proliferation, angiogenesis, invasion, metastasis as well as chemo- and radio-resistance [35]. While in majority of cases, NF-κB serves as a transcriptional activator by binding to the promoter of genes to enhance gene transcription, which holds accountable for most biochemical and biological functions, including tumor growth [12]. After determining the direct binding relationship between NF-κB and CCND1, we found the interaction was dependent on NF-κB nuclear translocation. Impairment of NF-κB nuclear translocation with the recombinant peptide SN50 has been proved to attenuate the elevation in CCND1 expression caused by N-methyl-D-aspartate receptor agonist quinolinic acid in striatal neurons [36], which was largely in line with what we observed in lung cancer cells.

To gain deeper insights into the downstream mechanism of CCND1, our study focused on the PI3K/AKT pathway with the help of KEGG enrichment analysis. The anti-tumor effects of ultrasound-targeted microbubble destruction-delivered si-CCND1 were elicited via blockage of the PI3K/AKT pathway in hepatocellular carcinoma [37]. In this study, Western blot was carried out to assess the expression of the PI3K/AKT pathway-related proteins. It was observed that CCND1-OE activated the PI3K/AKT signaling, while inhibition of NF-κB using SN50 reversed the stimulative effects, which has rarely been reported and highlighted the novelty of our study. Later, rescue experiments using SN50 and the PI3K/AKT signaling inhibitor PI3K-IN-1 revealed that suppression of NF-κB or impairment of the PI3K/AKT signaling could effectively counteract the oncogenic role of CCND1 in lung cancer *in vitro* and *in vivo*. Interestingly, cervix cancer-derived cells treated with the PI3K inhibitor LY294004 and the proteasome inhibitor MG132 led to induced a G1 and a G2/M cell cycle arrest, respectively [38], signifying that the PI3K/AKT signaling is also linked to cell cycle progression in cancers.

## Conclusion

In conclusion, we found that CCND1 is increased in lung cancer in a NF-κB nuclear translocation-dependent manner, which promotes lung cancer cell proliferation and oncogenic activity by inducing the PI3K/AKT signaling pathway. Our results propose that inhibition of CCND1 might be an attractive therapeutic target for lung cancer. In the future, more efforts should be devoted to outline the mechanisms of CCND1 as a tumor oncogene in cancers.

## Data Availability

The data used to support the findings of this study are included within the article.
